# Carbon-free energetic materials: computational study on nitro-substituted BN-cage molecules with high heat of detonation and stability†

**DOI:** 10.1039/c7ra13476b

**Published:** 2018-04-18

**Authors:** Xin Zeng, Nan Li, Qingjie Jiao

**Affiliations:** State Key Laboratory of Explosion Science and Technology, School of Mechatronical Engineering, Beijing Institute of Technology Beijing 100081 China leen04@bit.edu.cn leen04@163.com jqj@bit.edu.cn jqjbit@126.com

## Abstract

A new series of high-energy density materials (HEDMs) B_6_N_6_H_6−*n*_(NO_2_)_*n*_ (*n* = 1–6) are studied at the M06-2X/6-311++G**, ωB97XD/6-311++G** and B3LYP/6-311++G** levels. Analysis of the structural changes caused by substituting the NO_2_ and the electronic structures, such as electron localization function (ELF), Wiberg bond index (WBI), charge transfer and bond dissociation energies (BDE), provide important insights into the essence of the chemical characteristics and stability. Moreover, the Born–Oppenheimer molecular dynamic (BOMD) simulation is performed to verify their stability, which suggests that only the BN-cage derivatives with one and two nitro groups bonding with boron atoms (NO_2_-1-1 and NO_2_-2-1) can remain stable under ambient conditions. To predict the detonation performance and sensitivity of these two stable BN-cage energetic molecules accurately, the density, gas phase enthalpy of formation, enthalpy of sublimation, detonation performance, impact sensitivity and BDE are calculated systematically. The calculation results show that both NO_2_-1-1 and NO_2_-2-1 have a higher heat of detonation, higher value of *h*_50_, and larger BDE of trigger bonds than CL-20.

## Abstract 2

A new series of high-energy density materials were created by replacing the H atoms in the BN-cage with nitro groups.

## Graphical abstract

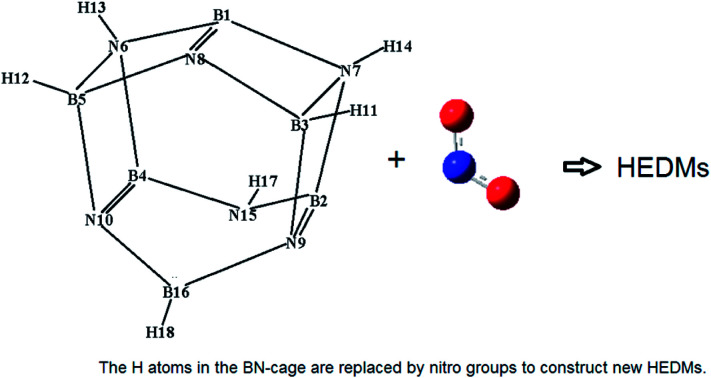

## 1. Introduction

High-energy density materials (HEDMs) with both superior detonation performance and low sensitivity have always drawn the attention of research scientists.^1–11^ The increased energy density often comes at the expense of molecular stability. Seeking new HEDMS with a fine balance between high detonation performance and low sensitivity remains to be an interesting, but very challenging task.

It is recognized that the best known explosive with superior detonation performance is 2,4,6,8,10,12-hexanitro-2,4,6,8,10,12-hexaazatetracyclo[5.5.0.0.0]dodecane (HNIW or CL-20), which was successfully synthesized by Nielsen in 1987.^12^ Besides the energetic functional groups, the large strain energy of its cage skeleton also allows CL-20 to store a high amount of energy. Currently, boron containing energetic materials have captured a great deal of attention in energetic materials research.^13–21^ The addition of boron in both micro and nano sizes into energetic materials can increase the energy output effectively and improve the detonation properties due to its high heat of combustion (36.3 kJ mol^−1^, 1.9 times than that of aluminum).^13–21^ Thus, from the point of view of energy release during combustion, boron- and nitrogen-containing complexes could be a good choice for developing high energy materials.

Inspired by this, we attempted to replace the C atoms of CL-20 with B atoms to develop a new carbon-free BN-cage energetic system. The nitro (NO_2_) groups are bonded to the BN-cage structure to increase the nitrogen and oxygen content, which can achieve a good oxygen balance. In this theoretical study, the BN-cage and its NO_2_ derivates are systematically investigated to understand their stability, electronic structures, safety and detonation properties at the M06-2X/6-311++G**, ωB97XD/6-311++G** and B3LYP/6-311++G** levels of density functional theory (DFT). Through the systemic study of the above compounds, it is predicted that two BN-cage derivatives, NO_2_-1-1 and NO_2_-2-1, will have a superior detonation performance and low sensitivity, and could therefore potentially be new HEDMs.

## 2. Theoretical methods

All quantum mechanical calculations in this paper were performed using the Gaussian-09 software package^22^ at the M06-2X, ωB97XD and B3LYP level with the 6-311++G** basis set. The M06-2X meta-hybrid density functional developed by Zhao and Truhlar is an advanced method to calculate energies, which have shown a good performance for main group chemistry and kinetics studies.^23,24^ For comparison, the theoretical methods of ωB97XD and B3LYP were also employed with the basis set of 6-311++G**. The ωB97XD method includes a 100% long-range exact exchange, a small fraction (about 22%) of the short-range exact exchange, a modified B97 exchange density functional for a short-range interaction, and the B97 correlation density functional and empirical dispersion correction.^25,26^ ωB97XD yields satisfactory accuracy for thermochemistry and kinetics. The DFT method of B3LYP combines Becke's three-parameter (B3)^27^ functional with the Lee–Yang–Parr (LYP)^28^ correlation functional, which has been considered to be capable of accurately predicting the structural parameters and frequencies of many nitro-substituted compounds.^29–31^ Moreover, the 6-311++G** basis set can generally give satisfactory geometries.^32^ All of the optimized molecular structures in this paper belong to local minima on their singlet spin state potential surface and have no imaginary frequency. Structures of the nitro-substituted BN-cage compounds are denoted as NO_2_-*n*-N in the present article, where *n* is the number of energetic groups, N orders the structures according to their relative energies using the M06-2X method.

Calculation of the electron localization function (ELF), deformation energies, net charge and charge transfer at the same level are used to analyze the electronic structure and stability of the NO_2_-*n*-N series. Moreover, Born–Oppenheimer molecular dynamic simulations (BOMD) are employed to exam the stability of the designed cage compounds to select the stable BN-cage derivates with a DFT in the level of M06-2X/6-311++G**. BOMD deals with the electronic and nuclear problems separately. In this method, the electronic structure in the ground state is calculated at each set of atomic positions, usually by optimization of the Kohn–Sham orbitals using an iterative method.^33^ The BOMD method in general needs more CPU time than for other MD software, but the method is more robust and stable.

Heat of detonation, detonation velocity and detonation pressure were calculated using EXPLO 5,^34^ using the density and solid phase enthalpy of formation (Δ_f_*H*°(s)). The gas phase enthalpy of formation (Δ_f_*H*°(g)) and the enthalpy of sublimation 
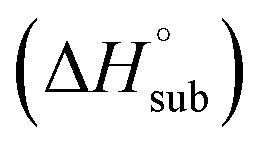
 were used to calculate the solid phase enthalpy of formation (Δ_f_*H*°(s)).

The following isodesmic reactions (1) combined with the computational Formula (2) were used to calculate the Δ_f_*H*°(g) of the studied compounds.1
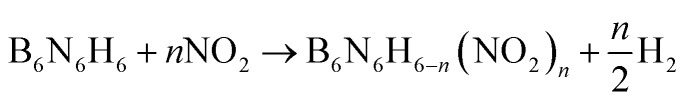

2 Δ*H*_298_ = Δ*E* + ΔZPE + Δ*H*_T_

In eqn (1) and (2), *n* is the number of nitro groups, Δ*H*_298_ is the enthalpy change of the reaction at 298 K, Δ*E* is the change in total energy between the products and the reactants, ΔZPE is the change of zero-point energy between the products and the reactants, and Δ*H*_T_ is the thermal correction from 0 to 298 K.

Electrostatic potential analysis (ESP) is employed to analyze the molecular surface at the M06-2X/6-311++G level of theory. In the eqn (3)–(7), the electrostatic potential on the surface is characterized by *V*_s_(*r*). *V*^+^_s_(*r*_*i*_), *V*^−^_s_(*r*_*i*_) and *V*_s_(*r*_*i*_) represents the positive, negative and the overall value of *V*_s_(r) at any point *r*_*i*_ on the surface. The 
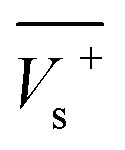
, 
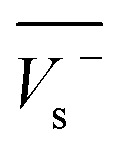
 and 
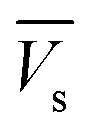
 represent their averages, and *σ*_+_^2^, *σ*_−_^2^, and *σ*_tot_^2^ are the corresponding variances. *ν* is the electrostatic balance parameter. *m* and *n* are the number of positions with positive and negative potentials on the molecular surface. The parameters mentioned above will be used to further calculate the enthalpy of sublimation, density and impact sensitivity in the corresponding equations.3
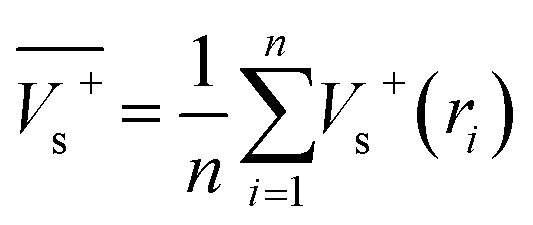
4
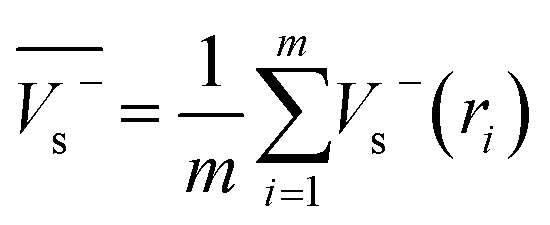
5
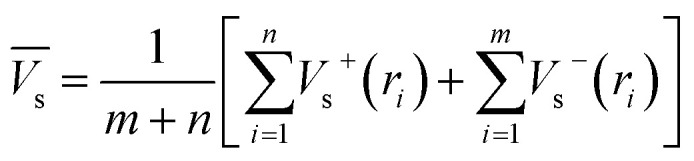
6
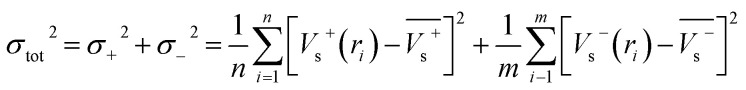
7
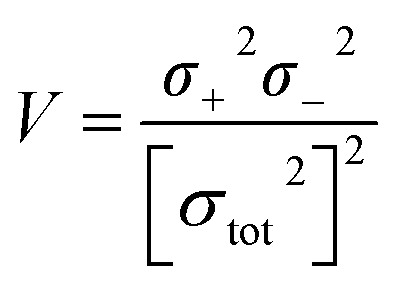


The enthalpy of sublimation 
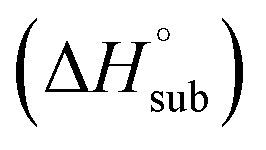
 can be evaluated using the developed method by Rice and Politzer *et al.*^35^8
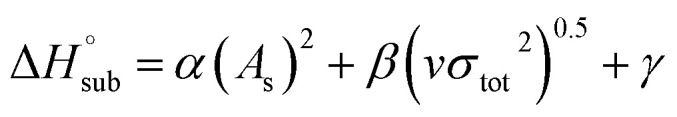
In eqn (8), *α* = 0.000267, *β* = 1.650087, and *γ* = 2.966078.

Then, the solid phase enthalpy of formation (Δ_f_*H*°(s)) can be calculated using eqn (9).9
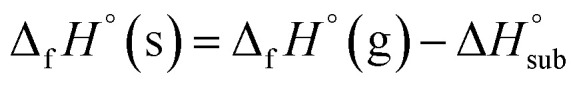


The crystal density (*ρ*) of the studied compounds can be calculated using 
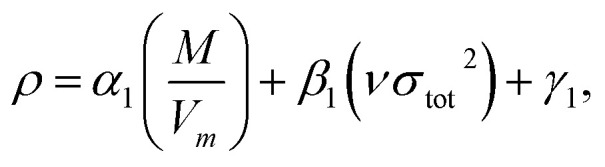
^36^ in which *α*_1_ = 0.9183, *β*_1_ = 0.0028, and *γ*_1_ = 0.0443.

In order to understand the sensitivity and safety of the studied cage compounds, the impact sensitivity,^37^ Wiberg bond index (WBI),^38,39^ and bond dissociation energy (BDE)^40^ were calculated. Impact sensitivity can be tested using the drop hammer test, which is measured by dropping a given mass (2.5 kg) upon the compound and recording the height (*h*_50_) which has a 50% probability of producing an explosion. The value of *h*_50_ is calculated using eqn (10).^37^

10 *h*_50_ = *α*_2_*σ*_+_^2^ + *β*_2_*ν* + *γ*_2_

In eqn (10), *α*_2_ = −0.0064, *β*_2_ = 241.42, and *γ*_2_ = −3.43.

The BDE is calculated using eqn (11). In general, if the value of BDE is greater than 84 kJ mol^−1^, the corresponding bond is considered to be stable.

11 BDE_(A–B)_ = [*E*_A˙_ + *E*_B˙_] − *E*_(A–B)_

## 3. Results and discussion

### 3.1. Configuration

**Fig. 1:**
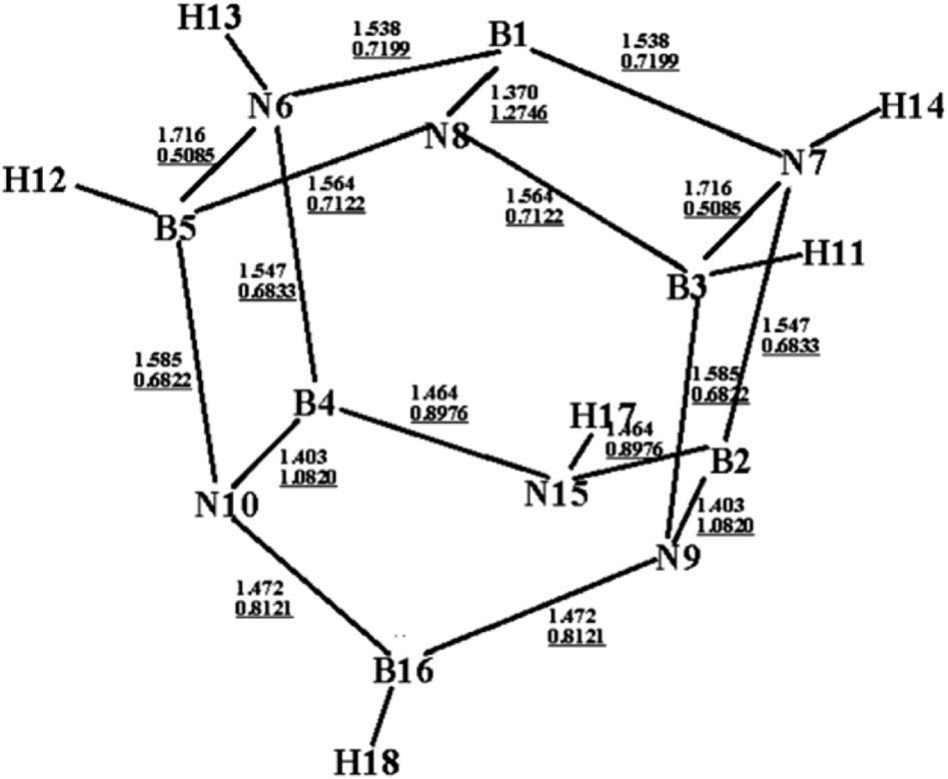
Optimized structure (bond lengths in Å) for the designed BN-cage at the M06-2X/6-311++G** level. Wiberg bond indexes are underlined.

Fig. 1 shows an optimized structure of the designed BN-cage that was confirmed to be a global minimal without imaginary frequencies at the M06-2X level. The distances of B3-N7, B5-N6 and B16-N15 are 1.716 Å, 1.716 Å and 2.803 Å, respectively. The corresponding WBI, determined using natural bond orbital (NBO) analysis were 0.5085, 0.5085 and 0.0049, respectively. The relatively long distance and small WBI between B16 and N15 suggests the absence of the corresponding bond. Additionally, the bond lengths (WBI) of B1–N8, B2–N9 and B4–N10 were found to be 1.370 Å (1.2746), 1.403 Å (1.0820) and 1.403 Å (1.0820), respectively. The shorter bond lengths and larger WBI of these three bonds compared with the other B–N bonds are indicative that double bonds exist between B1–N8, B2–N9 and B4–N10. Also, the topological analysis of the ELF was performed to understand the bond character of the B–N bonds in the designed BN-cage. The corresponding color-filled maps and the curve maps of ELF for these bonds are shown in Fig. S1,† which further confirms the existence of the B3–N7, B5–N6, B1–N8, B2–N9, and B4–N10 covalent bonds, and the inexistence of the B16–N15 bond.

**Fig. 2:**
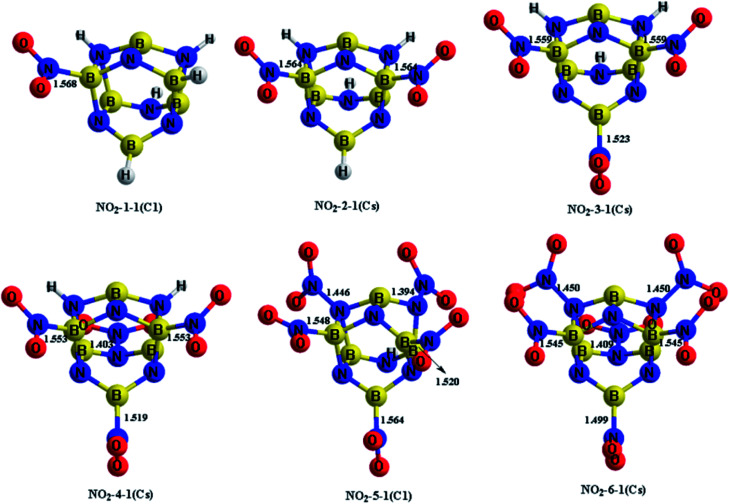
Optimized geometries (bond lengths in Å) at the M06-2X/6-311++G** level for the NO_2_-*n*-1 (*n* = 1–6). Symmetries are labeled in parentheses.

The H atoms in the designed BN-cage were replaced by nitro groups to construct a series of new energetic molecules. All of the optimized structures could retain the integrity of the BN-cages. Moreover, the calculation results indicated that the energetic groups bond with boron atoms in the cage led to lower-energy structures than that with nitrogen atoms. Fig. 2 shows the optimized geometries of the NO_2_-*n*-1 (*n* = 1–6).

The four mononitro-substituted BN-cage compounds shown in Fig. S1 and Table S2† were found to lie less than 200 kJ mol^−1^ in energy above the lowest-energy structure NO_2_-1-1. The nitro group in the lowest-lying structures NO_2_-1-1 is bonded to the B5 atom by replacing H12 in the BN-cage. It should be pointed out that the distance between B3 and N7 in NO_2_-1-3 is 2.621 Å, the long distance of the bond and the corresponding ELF analysis (shown in Fig. S3†) suggests that the B3–N7 bond does not exist.

The five dinitro-substituted BN-cage compounds shown in Fig. S2 and Table S3† are found to lie less than 200 kJ mol^−1^ in energy above NO_2_-2-1. Similar to the mononitro-substituted compounds, the nitro groups bond with B3 and B5 in NO_2_-2-1 to obtain the lowest-energy structure with the symmetry of the *C*_s_. The NO_2_-2-2 lies at an energy of 27.2 kJ mol^−1^ above the NO_2_-2-1S at the M06-2X level, having the B5–NO_2_ and B16–NO_2_ bonds. However, the NO_2_-2-3, NO_2_-2-4 and NO_2_-2-5, with the N-NO_2_ bonds have much higher total energies than the NO2-2-1 and NO2-2-2 due to the high energy of the N–N bonds.

The four trinitro-substituted BN-cage compounds shown in Fig. S3 and Table S4† are found to lie less than 200 kJ mol^−1^ in energy above the lowest-energy structure NO_2_-3-1. The three nitro groups in NO_2_-3-1 with a *C*_s_ symmetry are bonded with the B3, B5 and B16 atoms. The NO_2_-3-2, NO_2_-3-3 and NO_2_-3-4 with one N–NO_2_ bond are, respectively, 126.0, 150.5 and 161.2 kJ mol^−1^ at the M06-2X level, higher than NO_2_-3-1.

As shown in Fig. S4 and Table S5,† three tetranitro-substituted BN-cages are found to lie less than 200 kJ mol^−1^ in energy above NO_2_-4-1. Both of the first two structures NO_2_-4-1 and NO_2_-4-2 have three B–NO_2_ bonds and one N–NO_2_ bonds, while the NO_2_ groups in NO_2_-4-3 bond with two B atoms and two N atoms. It should be pointed out that the distances between the B3 and N7 atoms in NO_2_-4-2 and NO_2_-4-3 are 2.534 and 2.491 Å, respectively. The relatively long distances and the corresponding ELF analysis shown in Fig. S7 and S8† suggests the absence of corresponding bonds.

Only the two pentanitro-substituted BN-cages were found, which are shown in Fig. S5 and Table S6.† In NO_2_-5-1, the five nitro groups bond with the three boron atoms and two nitrogen atoms. Additionally, the distance of B3⋯N7 and B16⋯N15 in NO_2_-5-1 are 2.608 and 2.629 Å respectively, which are about 1 Å longer than the other B–N bonds. This is indicative of the absence of these two bonds, combined with the color-filled map and the curve map of ELF (shown in Fig. S10†).

Finally, only one hexanitro-substituted BN-cage NO_2_-6-1 is found, as shown in Fig. S6 and Table S7.† All of the H atoms in the designed BN-cage are replaced by nitro groups leading to the *C*_s_ symmetry for NO_2_-6-1.

### 3.2. Electronic structure and thermal stability

To study the change caused by substituting the H of the designed BN-cage with NO_2_, the deformation energies of the skeleton (Δ*E*_cage_) and the relative (ΔBDE) are calculated and summarized in Table 1. ΔBDE can be calculated using eqn (12), which is the relative BDE of cage-NO_2_ bonds to the corresponding cage-H bonds in the designed BN-cage without substitution.

12 ΔBDE = BDE_cage–NO_2__ − BDE_cage–H_

Δ*E*_cage_ corresponds to the change of the BN-cage skeleton. The Δ*E*_cage_ shown in Table 1 indicates that the substitution of the nitro groups leads to the deformation of the cage skeleton. Adding nitro groups to the nitrogen atoms of the BN-cage brings more changes to the skeleton compared with the boron atoms. It should be pointed out that NO_2_-1-3 has the most skeleton changes among the four isomers, caused by the breaking of the B3–N7 bond. Negative values of ΔBDE suggest that the bond strengths of all the cage-NO_2_ bonds are lower than that of the corresponding cage-H bonds.

**Table 1:** The deformation energies of the BN-cage skeleton (Δ*E*_cage_, kJ mol^−1^) and the relative BDE (ΔBDE, kJ mol^−1^) to the designed BN-cage without substitution

Compounds	Methods	NO_2_-1-1	NO_2_-1-2	NO_2_-1-3	NO_2_-1-4
Δ*E*_cage_	M06-2X	0.5	1.6	53.4	44.4
	ωB97XD	2.83	4.0	54.0	43.4
	B3LYP	3.9	1.6	55.5	30.5
ΔBDE	M06-2X	−24.6	−55.4	−212.8	−226.8
	ωB97XD	−39.5	−62.8	−226.2	−241.4
	B3LYP	−37.0	−61.0	−225.5	−228.2

To further study the stability of the mononitro-substituted BN-cage isomers, the deformation energies of the BN-cage skeletons 
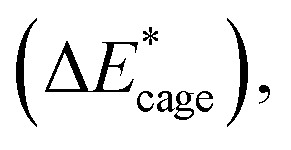
 the deformation energies of the nitro groups 
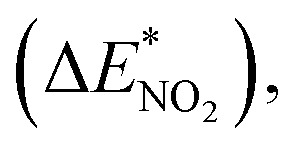
 and the relative BDE of the cage-NO_2_ bonds (ΔBDE*) to NO_2_-1-1 are calculated and the results are shown in Table 2.

**Table 2:** The relative energies (Δ*E*, kJ mol^−1^), relative BDE (ΔBDE*, kJ mol^−1^), and the deformation energies of the BN-cage skeletons 
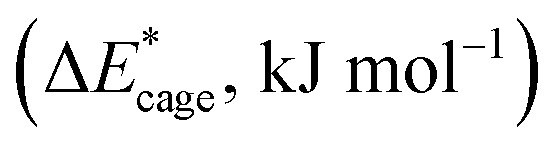
 and nitro groups 
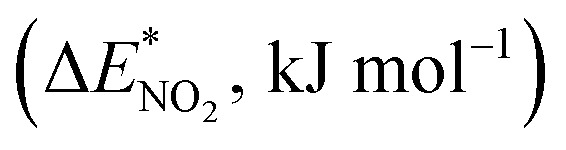
 among the mononitro-substituted BN-cage compounds

Compounds	Methods	NO_2_-1-1	NO_2_-1-2	NO_2_-1-3	NO_2_-1-4
Δ*E*	M06-2X	0	24.6	129.7	151.3
	ωB97XD	0	20.3	124.7	149.4
	B3LYP	0	17.2	126.0	152.4
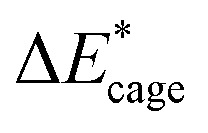	M06-2X	0	−0.2	−26.0	−18.9
	ωB97XD	0	−0.2	−45.7	−35.8
	B3LYP	0	−1.3	−50.2	−48.1
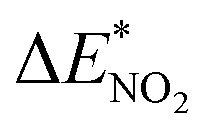	M06-2X	0	−4.2	−4.7	−6.1
	ωB97XD	0	−4.3	−5.2	−6.5
	B3LYP	0	−3.2	−3.0	−4.5
ΔBDE*	M06-2X	0	−29.0	−160.4	−176.2
	ωB97XD	0	−24.7	−175.6	−191.6
	B3LYP	0	−22.9	−179.3	−204.9

As shown in Table 2, comparing with NO_2_-1-1, the decrease of the BDE of the B–NO_2_ bond in the NO_2_-1-2 structure leads to an increase of its energy, and the deformation of its skeleton could be ignored due to its small 
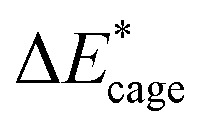
 value. For the N-mononitrosubstituted isomers NO_2_-1-3 and NO_2_-1-4, the difference of the B–NO_2_ and N–NO_2_ bond is the primary reason leading to their energy increase. Compared to the global minimum of NO_2_-1-1, the NO_2_ bonding in the N atom brings the relative heavy deformation of the BN-cage skeletons, which is another reason for the lower NO_2_-1-3 and NO_2_-1-4 structural stability. For the nitro group, the isomers have a small 
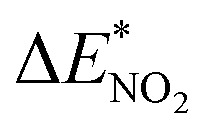
 value and the change of the NO_2_ could be ignored, as shown in Table 2.

**Fig. 3:**
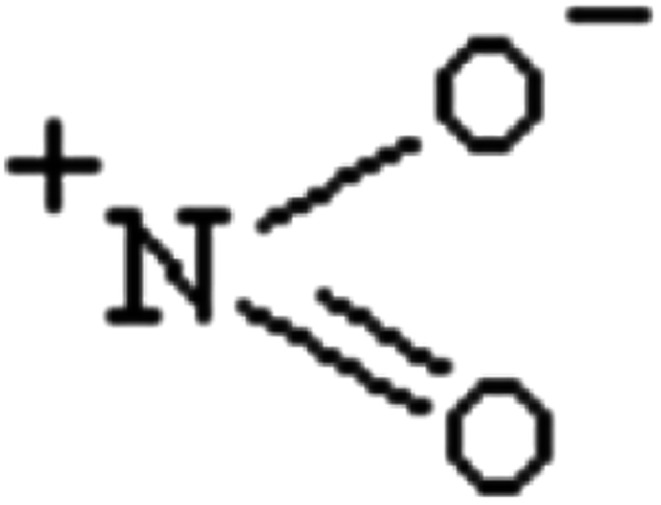
The valence-bond structures of the nitro group.

**Fig. 4:**
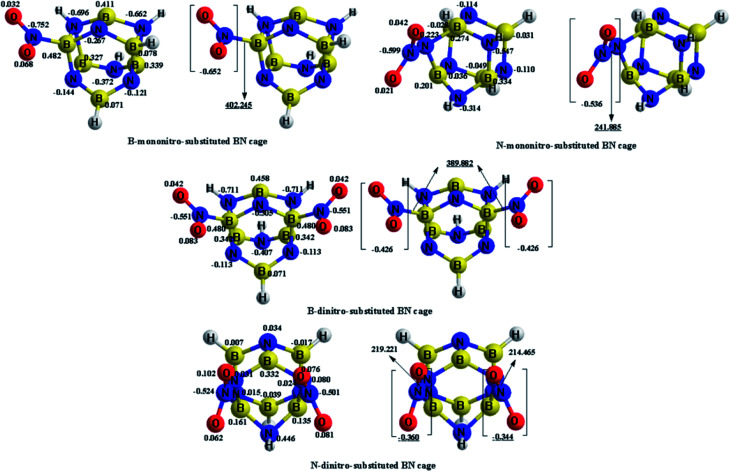
Optimized geometries, net charge (*e*), and charge transfer (*e*) of the B(N)-mononitro(dinitro)-substituted BN-cage compounds at the M06-2X/6-311++G** level. The BDE (kJ mol^−1^) of the cage-NO_2_ bonds are underlined.

The charge transfer calculated using the NBO analysis is also employed to analyze the stability differences among the different substitution positions. The valence-bond structure of the nitro group, shown in Fig. 3, indicates that the nitro is an electrophilic group, in which the nitrogen atom tends to combine with the boron atom in the cage to provide a stable configuration. Fig. 4 and Table 3 describe the molecular structures, net charge, charge transfer and BDE of the cage-NO_2_ bonds for the mononitro or dinitro-substituted cages. As listed in Table 3, the results show that all the nitro groups in the B(N)-mononitro or dinitro-substituted cages are electron accepters, and the charge transfers of the B-mononitro or dinitro-substituted cage is always larger than those of the N-mononitro or dinitro-substituted cage, suggesting the higher dissociation energies of the B–NO_2_ bonds compared with the N–NO_2_ bonds.

**Table 3:** The net charge (*e*) on the nitro groups in the B(N)-mononitro(dinitro)-substituted BN-cage compounds at the M06-2X/6-311++G**, ωB97XD/6-311++G**, and B3LYP/6-311++G** levels

Compounds	Substitution positions	Net charge		
		M06-2X	ωB97XD	B3LYP
B-mononitro-substitution	B	−0.652	−0.627	−0.740
N-mononitro-substitution	N	−0.536	−0.497	−0.504
B-dinitro-substitution	B	−0.426	−0.388	−0.696
	B	−0.426	−0.388	−0.696
N-dinitro-substitution	N	−0.360	−0.322	−0.349
	N	−0.344	−0.317	−0.349

The above calculations suggest that the B-nitrosubstituted cages have a relatively better stability than the N-nitrosubstituted cages. As such, the NO_2_-1-1, NO_2_-2-1 and NO_2_-3-1 structures were selected for further study, as discussed in the subsequent sections.

### 3.3. Dynamic simulation

Based on the above calculation results for the stability of these designed BN-cage derivatives, molecular dynamic calculations of the B-substituted NO_2_-*n*-1 (*n* = 1, 2, 3) structures were carried out to exam their dynamic stability. BOMD were employed to perform the molecular dynamic simulation. The static configurations calculated at the M06-2X/6-311++G** level were run as seeds for the BOMD trajectories using the Gaussian-09 program package. Equilibration steps were performed over 1 ps and production runs are 3 ps long with the time step of 1 fs.

**Fig. 5:**
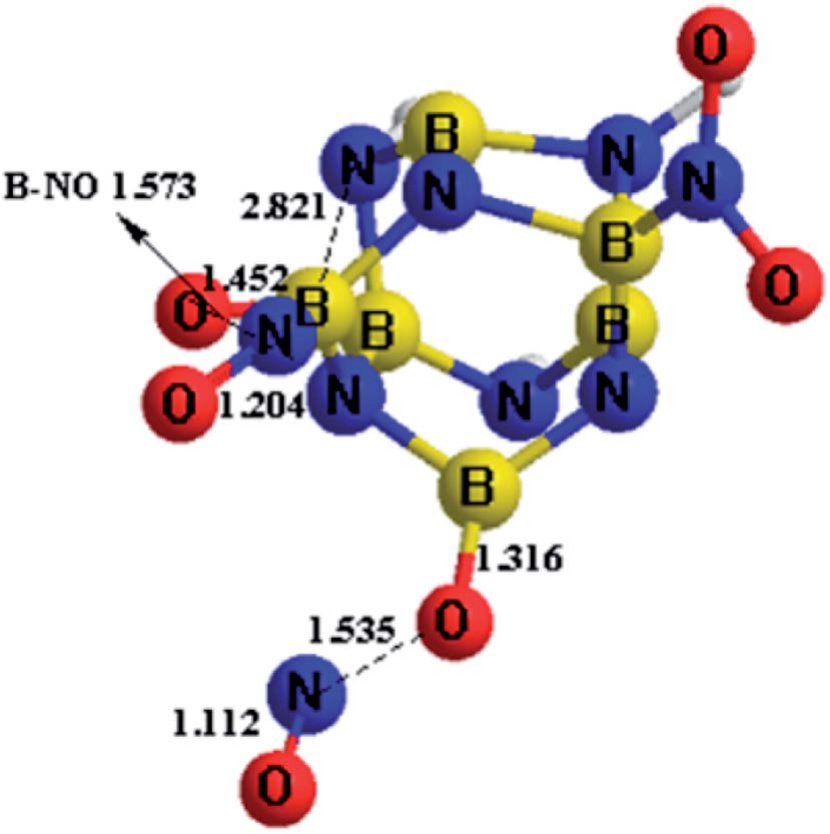
The dynamic simulation results of NO_2_-3-1 at 298 K, taken from the simulations performed at the M06-2X/6-311++G** level.

The simulation results of the NO_2_-*n*-1 (*n* = 1, 2, 3) structures show that the NO_2_-1-1 and NO_2_-2-1 remain stable at 298 K during the whole simulation, while the nitro groups in NO_2_-3-1 cannot remain stable at 298 K, as shown in Fig. 5. As can be seen from Fig. 5, the nitro groups in NO_2_-3-1 decompose from the cage with the cage-NO_2_ bond breaking. Oxygen atoms in the nitro groups in the NO_2_-3-1 bond with the cage directly at 298 K, with B–O bond lengths of 1.452 and 1.316 Å, while the NO groups decompose from the BN-cage compounds, with the distances between O and NO groups being ∼1.55 Å. Moreover, deformation of the cage skeleton occurs with the distance between B5 and N6 being 2.821 Å. Thus, the NO_2_-3-1 structure cannot remain stable at room temperature. Above all, the NO_2_-1-1 and NO_2_-2-1 structures have exhibited an attractive potential for application in the areas of energetic materials and the details are discussed in the following study on detonation performance.

### 3.4. Detonation performance

In order to quantitatively evaluate the detonation performance of the NO_2_-1-1 and NO_2_-2-1 structures, the predicted density (*ρ*), enthalpy of sublimation 
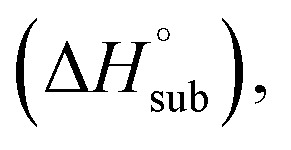
 solid phase enthalpy of formation (Δ_f_*H*°(s)), oxygen balance and detonation parameters (*Q*, *D*, *P*) were calculated systematically using EXPLO 5. The results, together with the related information for CL-20, are listed in Table 4 for comparison.

**Table 4:** Theoretical density (*ρ*), enthalpy of sublimation 
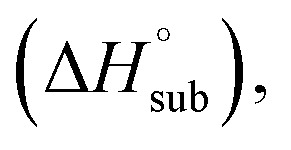
 solid phase enthalpy of formation (Δ_f_*H*°(s)), detonation velocity (*D*), detonation pressure (*P*), heat of detonation (*Q*) and oxygen balance of the NO_2_-1-1 and NO_2_-2-1 structures. Experimental parameters of CL-20 are labeled in parentheses (the theoretical densities of CL-20 are 1.954 g cm^−3^ at M06-2X, 1.907 g cm^−3^ at ωB97XD and 2.022 g cm^−3^ at B3LYP)

compounds	NO_2_-1-1			NO_2_-2-1			CL-20
Methods	M06-2X	ωB97XD	B3LYP	M06-2X	ωB97XD	B3LYP	
*ρ* (g cm^−3^)	1.808	1.789	1.732	1.931	1.871	2.059	(2.04)
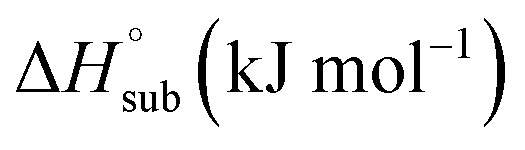	124.900	123.909	125.908	132.842	134.435	137.619	—
Δ_f_*H*°(s) (kJ mol^−1^)	−289.545	−269.297	−274.313	−449.511	−413.859	−554.623	—
*D* (m s^−1^)	6485.349	6409.758	6132.865	7222.655	6881.267	7019.696	(9580)
*P* (GPa)	15.449	14.783	13.427	24.959	18.518	26.566	(43.2)
*Q* (kJ kg^−1^)	8131.956	8180.25	8115.766	8287.309	8287.938	7879.410	(6314)
Oxygen balance	−76.011%			−45.720%			−10.953%

The results in Table 4 show that the *ρ* of NO_2_-1-1 and NO_2_-2-1 are 1.808 g cm^−3^ (1.789 g cm^−3^, 1.732 g cm^−3^) and 1.931 g cm^−3^ (1.871 g cm^−3^, 2.059 g cm^−3^) respectively at the M06-2X (ωB97XD, B3LYP)/6-311++G** level. In the meantime, the calculated densities of CL-20 are 1.954 g cm^−3^ at the M06-2X level, 1.907 g cm^−3^ at the ωB97XD level and 2.022 g cm^−3^ at the B3LYP level, which indicates the relatively high densities of these two newly proposed designed molecules. The *Q* of NO_2_-2-1 is 31.25% (M06-2X), 31.26% (ωB97XD) or 24.79% (B3LYP) higher than that of CL-20 (6314 kJ kg^−1^), with a *D* of over 6800 m s^−1^. The superior detonation heat of both the NO_2_-1-1 and NO_2_-2-1 structures indicate the great potential of these two energetic compounds to be HEDMs.

### 3.5. Sensitivity and safety

To test the sensitivity and safety of the above NO_2_-1-1 and NO_2_-2-1 structures, two methods have been employed in the present work: (1) impact sensitivity; and (2) BDE of the trigger bonds.

#### 3.5.1 Impact sensitivity

The impact sensitivity is a crucial parameter responsible for effectiveness. It reflects whether the explosive is stable enough to be handled or stored under typical conditions. In this paper, the theoretical impact sensitivity of NO_2_-1-1, NO_2_-2-1 and CL-20 were calculated at the M06-2X, ωB97XD and B3LYP level to compare their safety and practicability (see Table 5). The calculation results show that the *h*_50_ of the NO_2_-1-1 and NO_2_-2-1 structures were 49.498 cm (47.745 cm, 50.555 cm) and 34.459 cm (34.089 cm, 36.115 cm) at the M06-2X (ωB97XD, B3LYP)/6-311++G** level, respectively, which are higher than that of CL-20. Such a result illustrates their high value of application.

**Table 5:** Calculated impact sensitivity (*h*_50_) of the predicted BN-cage compounds and CL-20. Experimental parameters of CL-20 are labeled in parentheses

Compounds	Methods	NO_2_-1-1	NO_2_-2-1	CL-20
*σ*_+_^2^	M06-2X	292.859	417.062	231.351
	ωB97XD	302.761	437.273	233.923
	B3LYP	278.559	422.418	306.392
*ν*	M06-2X	0.227	0.168	0.068
	ωB97XD	0.220	0.167	0.065
	B3LYP	0.231	0.175	0.058
*h*_50_ (cm)	M06-2X	49.498	34.459	11.506
	ωB97XD	47.745	34.089	10.765
	B3LYP	50.555	36.115	8.611

#### 3.5.2 Bond dissociation energy

The BDE of the trigger bond is often a key factor in investigating the thermal stability and pyrolysis mechanism of energetic compounds. Generally, the smaller the energy needed for breaking a bond, the weaker the bond.

To elucidate the pyrolysis mechanism and thermal stability of the NO_2_-1-1 and NO_2_-2-1 structures, the bond dissociation energies of the cage-NO_2_ bonds in NO_2_-1-1, NO_2_-2-1 and CL-20, as well as the corresponding WBI have been investigated in the present work, as shown in Table 6. Obviously, the BDE of the cage-NO_2_ bonds in the NO_2_-1-1 and NO_2_-2-1 structures are 402.245 kJ mol^−1^ (447.635 kJ mol^−1^, 393.573 kJ mol^−1^) and 389.882 kJ mol^−1^ (358.869 kJ mol^−1^, 381.905 kJ mol^−1^) with WBI of 0.6640 (0.6657, 0.6852) and 0.6716 (0.6731, 0.6916) respectively, at the M06-2X (ωB97XD, B3LYP)/6-311++G** level. Thus, the designed NO_2_-1-1 and NO_2_-2-1 BN-cage molecules meet the stability requirement suggested previously, that is, a molecule could be considered as a practical energetic material if it has its BDE larger than 84 kJ mol^−1^. Moreover, the BDE of the cage-NO_2_ bonds in NO_2_-1-1 and NO_2_-2-1 are much higher than that of CL-20 at the same calculation level, which implies that they have a better stability than CL-20.

**Table 6:** Calculated bond dissociation energies (BDE) and Wiberg bond index (WBI) of the trigger bonds (cage-NO_2_ bonds) in NO_2_-1-1 and NO_2_-3-1 at the M06-2X, ωB97XD and B3LYP level

Compounds	WBI			BDE(kJ mol^−1^)		
Methods	M06-2X	ωB97XD	B3LYP	M06-2X	ωB97XD	B3LYP
NO_2_-1-1	0.6640	0.6657	0.6852	402.245	447.635	393.573
NO_2_-2-1	0.6716	0.6731	0.6916	389.882	358.869	381.905
CL-20	0.9421	0.9460	0.9318	153.092	127.022	148.656

## 4. Conclusions

Inspired by the high combustion heat of the boron atom and the large strain energy of the HNIW cage skeleton, we proposed several molecules with a new BN-cage skeleton by replacing C with B atoms in this study. A series of energetic molecules B_6_N_6_H_6−*n*_(NO_2_)_*n*_ (*n* = 1–6) were subsequently constructed through systematically substituting NO_2_ groups for the H atoms of the BN-cage. Then, all the B_6_N_6_H_6−*n*_(NO_2_)_*n*_ (*n* = 1–6) structures were optimized at the M06-2X/6-311++G**, ωB97XD/6-311++G** and B3LYP/6-311++G** level of DFT. Analysis of the structural changes caused by substituting the NO_2_ and the electronic structures including ELF, WBI, charge transfer and BDE reveal the essence of the stability and chemical characteristics of these B_6_N_6_H_6−*n*_(NO_2_)_*n*_ (*n* = 1–6) molecules. The results show that these B-nitro substituted cages have a higher stability than the N-nitro substituted cages. The corresponding molecular dynamic simulations, which were performed to test the stability of NO_2_-1-1, NO_2_-2-1 and NO_2_-3-1, indicate that only the NO_2_-1-1 and NO_2_-2-1 structures remain stable under ambient conditions. Then, the density, detonation performance and safety of the NO_2_-1-1 and NO_2_-2-1 structures were calculated systematically. The results show that the two BN-cage energetic molecules have a much higher detonation heat than traditional energetic materials such as CL-20, due to their high densities and the particular characteristics of the “BN” groups. Calculations also showed much higher *h*_50_ values of NO_2_-1-1 (49.489 cm at the M06-2X level) and NO_2_-2-1 (34.459 cm at the M06-2X level) than CL-20 (11.506 cm at the M06-2X level) in the same level of calculation, as well as the high bond dissociation energies over 84 kJ mol^−1^. Above all, a new type of energetic material based on the BN-cage was put forward and could be a new field of research to find more HEDMs with a superior performance.

## Conflicts of interest

There are no conflicts of interest to declare.

## Supplementary Material

RA-008-C7RA13476B-s001
