# Supplementary material for: Carbon-free energetic materials: computational study on nitro-substituted BN-cage molecules with high heat of detonation and stability
Source: RSC Adv. 2018 Apr 18;8(26):14654–62. doi: 10.1039/c7ra13476b (PMC9079922; doi:10.1039/c7ra13476b)
Supplement: RA-008-C7RA13476B-s001 [file RA-008-C7RA13476B-s001.pdf]

Electronic Supplementary Material (ESI) for RSC Advances.
This journal is © The Royal Society of Chemistry 2018

Carbon-Free Energetic Materials: Computational Study on Nitro-substituted BN-cage Molecules with High Heat of Detonation and Stability

Xin Zeng, Nan Li*, Qingjie Jiao*

State key Laboratory of Explosion Science and Technology, School of Mechatronical Engineering, Beijing Institute of Technology, Beijing 100081, China

E-mail addresses: leen04@bit.edu.cn, leen04@163.com (Nan Li)

jqj@bit.edu.cn, jqjbit@126.com (Qingjie Jiao)

Table S1 The calculated densities for some compounds at the M06-2X/6-311++G** level with their experimental data

Figure S1 The color-filled map and curve map of ELF for B-N bond paths of the designed BN-cage at the M06-2X levels of theory

Figure S2 Optimized geometries (bond lengths in Å) at the M06-2X level for the mononitro-substituted BN-cage compounds and their isomers. Relative energies (kJ mol^{-1}) at M06-2X level are indicated in parentheses

Table S2 Total energies (E in hartree), relative energies (ΔE in kJ/mol) and symmetries for the mononitro-substituted BN-cage compounds and their isomers at the M06-2X,and B3LYP level.

Figure S3 The color-filled map and curve map of ELF for B3-N7 bond path of NO_2-1-3 at the M06-2X levels of theory

Figure S4 Optimized geometries (bond lengths in Å) at the M06-2X level for the dinitro-substituted BN-cage compounds and their isomers. Relative energies (kJ mol^{-1}) at M06-2X level are indicated in parentheses

Table S3 Total energies (E in hartree), relative energies (ΔE in kJ/mol) and symmetries for the dinitro-substituted BN-cage compounds and their isomers at the M06-2X, ωB97XD and B3LYP level.

Figure S5 Optimized geometries (bond lengths in Å) at the M06-2X level for the trinitro-substituted BN-cage compounds and their isomers. Relative energies (kJ mol^{-1}) at M06-2X level are indicated in parentheses

Table S4 Total energies (E in hartree), relative energies (ΔE in kJ/mol) and symmetries for the trinitro-substituted BN-cage compounds and their isomers at the M06-2X, ωB97XD and B3LYP level.

Figure S6 Optimized geometries (bond lengths in Å) at the M06-2X level for the tetranitro-substituted BN-cage compounds and their isomers. Relative energies (kJ mol^{-1}) at M06-2X level are indicated in parentheses

Figure S7 The color-filled map and curve map of ELF for B3-N7 bond path of NO_2-4-2 at the M06-2X levels of theory

Figure S8 The color-filled map and curve map of ELF for B3-N7 bond path of NO_2-4-3 at the M06-2X levels of theory

Table S5 Total energies (E in hartree), relative energies (ΔE in kJ/mol) and symmetries for the tetranitro-substituted BN-cage compounds and their isomers at the M06-2X, ωB97XD and B3LYP level.

Figure S9 Optimized geometries (bond lengths in Å) at the M06-2X level for the pentanitro-substituted BN-cage compounds and their isomers. Relative energies (kJ mol^{-1}) at M06-2X level are indicated in parentheses

Table S6 Total energies (E in hartree), relative energies (ΔE in kJ/mol) and symmetries for the pentanitro-substituted BN-cage compounds and their isomers at the M06-2X, ωB97XD and B3LYP level.

Figure S10 The color-filled map and curve map of ELF for B3-N7 bond path of NO_2-5-1 at the M06-2X levels of theory

Figure S11 Optimized geometries (bond lengths in Å) at the M06-2X level for the hexanitro-substituted BN-cage compounds and their isomers. Relative energies (kJ mol^{-1}) at M06-2X level are indicated in parentheses

Table S7 Total energies (E in hartree), relative energies (ΔE in kJ/mol) and symmetries for the hexanitro-substituted BN-cage compounds and their isomers at the M06-2X, ωB97XD and B3LYP level.

Table S8 Total energies (E), zero point energies (ZPE), thermal correction values (H_T), heat of formation (kj/mol), molecular surface properties(As, vϭtot2), enthalpy of sublimation ($\Delta H°_{sub}$) and solid phase enthalpy of formation ($\Delta_f H°(s)$) of the NO_2-substituted BN-cage compounds at the M06-2X /6-311++G** level.

Table S9 Total energies (E), zero point energies (ZPE), thermal correction values (H_T), heat of formation (kj/mol), molecular surface properties(As, vϭtot2), enthalpy of sublimation ($\Delta H°_{sub}$) and solid phase enthalpy of formation ($\Delta_f H°(s)$) of the NO_2-substituted BN-cage compounds at the ωB97XD/6-311++G** level.

Table S10 Total energies (E), zero point energies (ZPE), thermal correction values (H_T), heat of formation (kj/mol), molecular surface properties(As, vϭtot2), enthalpy of sublimation ($\Delta H°_{sub}$) and solid phase enthalpy of formation ($\Delta_f H°(s)$) of the NO_2-substituted BN-cage compounds at the B3LYP/6-311++G** level.

Table S1 The calculated densities for some compounds at the M06-2X/6-311++G** level with their experimental data

compounds	Experiment(g/cm^3)	Calculated(g/cm^3)
TNT	1.654	1.647
RDX	1.816	1.800
HMX	1.902	1.877
PETN	1.732	1.656
CL-20	2.040	1.954

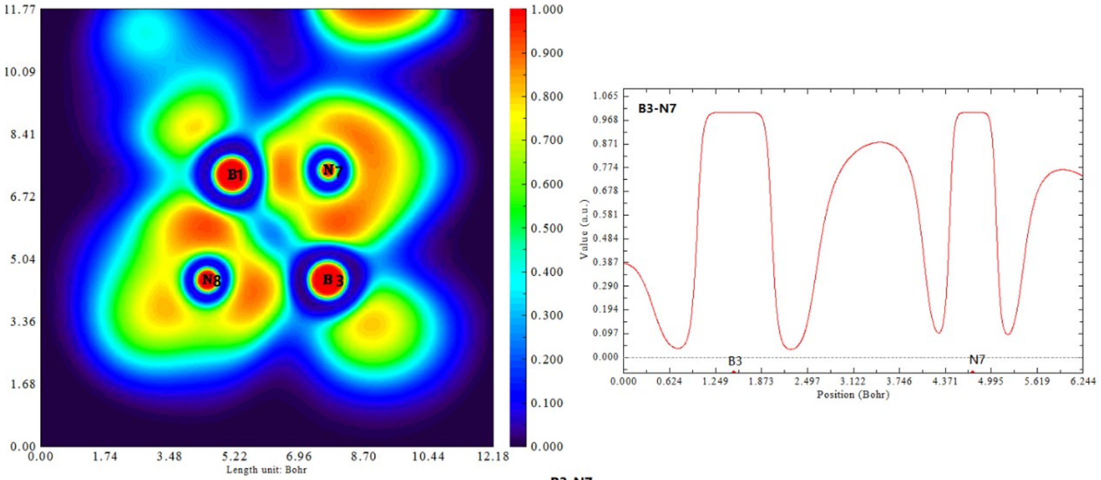

B3-N7

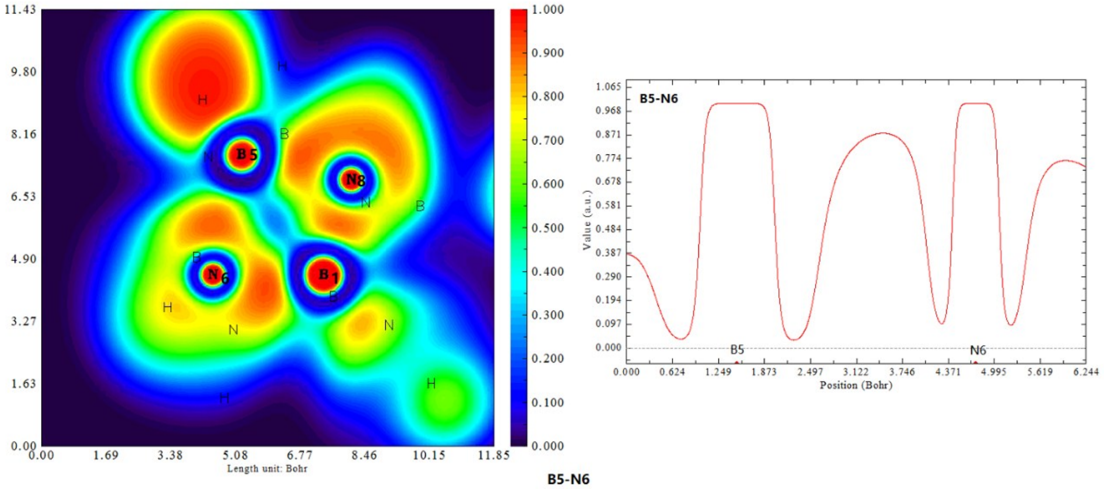

B5-N6

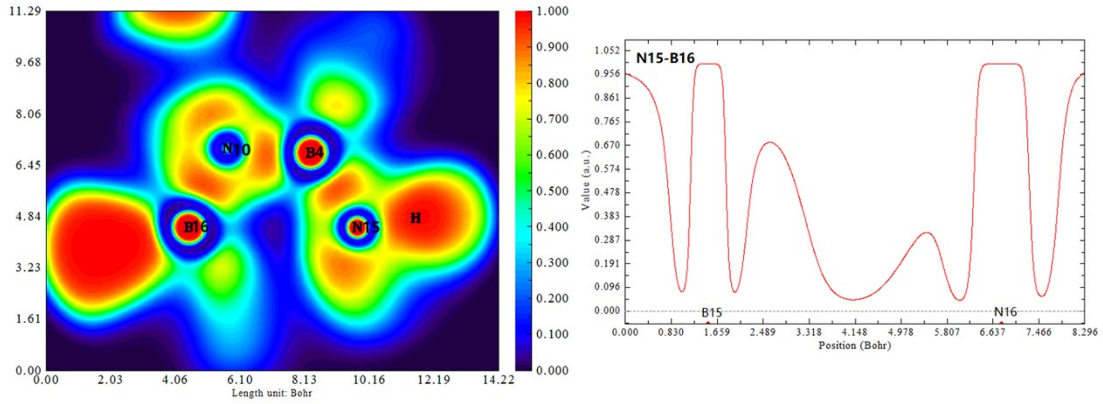

N15-B16

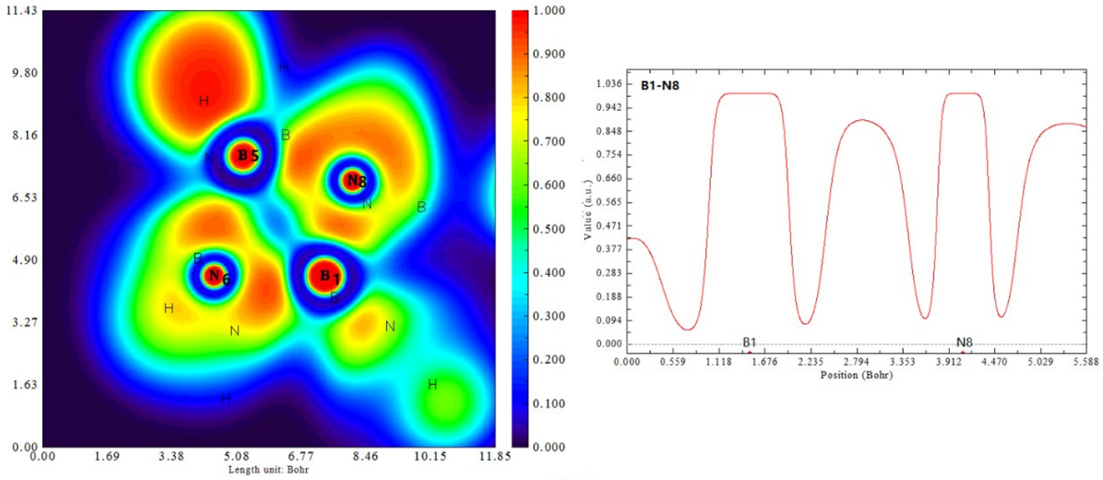

B1-N8

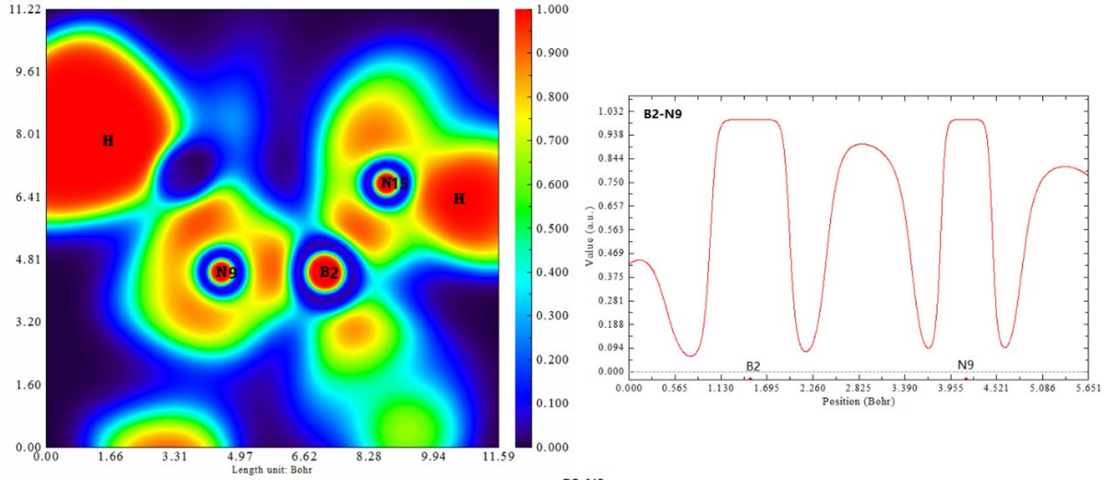

B2-N9

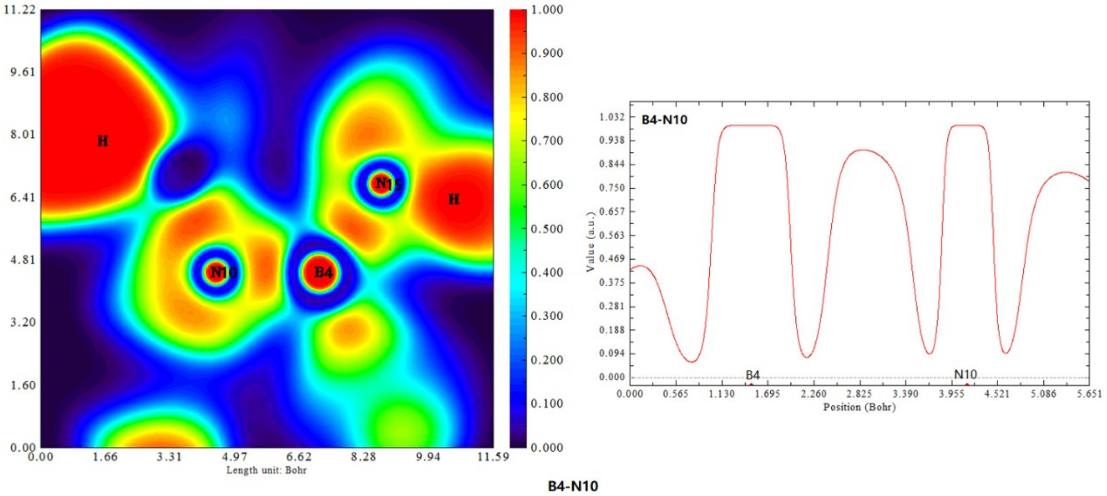

Figure S1 The color-filled maps and curve maps of ELF for B-N bond paths of the designed BN-cage at the M06-2X levels of theory

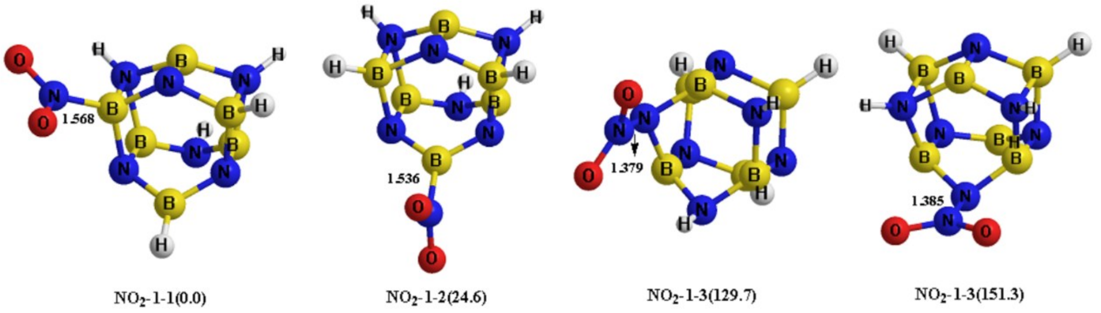

Figure S2 Optimized geometries (bond lengths in Å) at the M06-2X level for the mononitro-substituted BN-cage compounds and their isomers. Relative energies (kJ mol^{-1}) at M06-2X level are indicated in parentheses

Table S2 Total energies (E in hartree), relative energies (ΔE in kJ/mol) and symmetries for the mononitro-substituted BN-cage compounds and their isomers at the M06-2X, ωB97XD and B3LYP level.

		1-1	1-2	1-3	1-4
M06-2X	E	-685.871906	-685.862523	-685.822520	-685.814304
	ΔE	0	24.6	129.7	151.3
	symmetry	C1	Cs	C1	Cs
ω B97XD	E	-685.870761	-685.863046	-685.823269	-685.813890
	ΔE	0	20.3	124.7	149.4
	symmetry	C1	Cs	C1	Cs
B3LYP	E	-685.868852	-685.862337	-685.820809	-685.810796
	ΔE	0.0	17.2	126.0	152.4
	symmetry	C1	Cs	C1	Cs

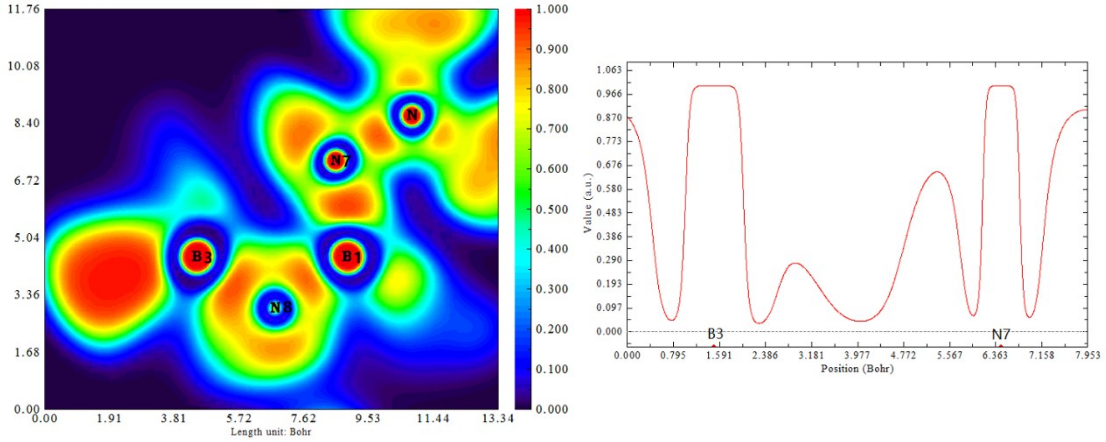

Figure S3 The color-filled map and curve map of ELF for B3-N7 bond path of NO_2-1-3 at the M06-2X levels of theory

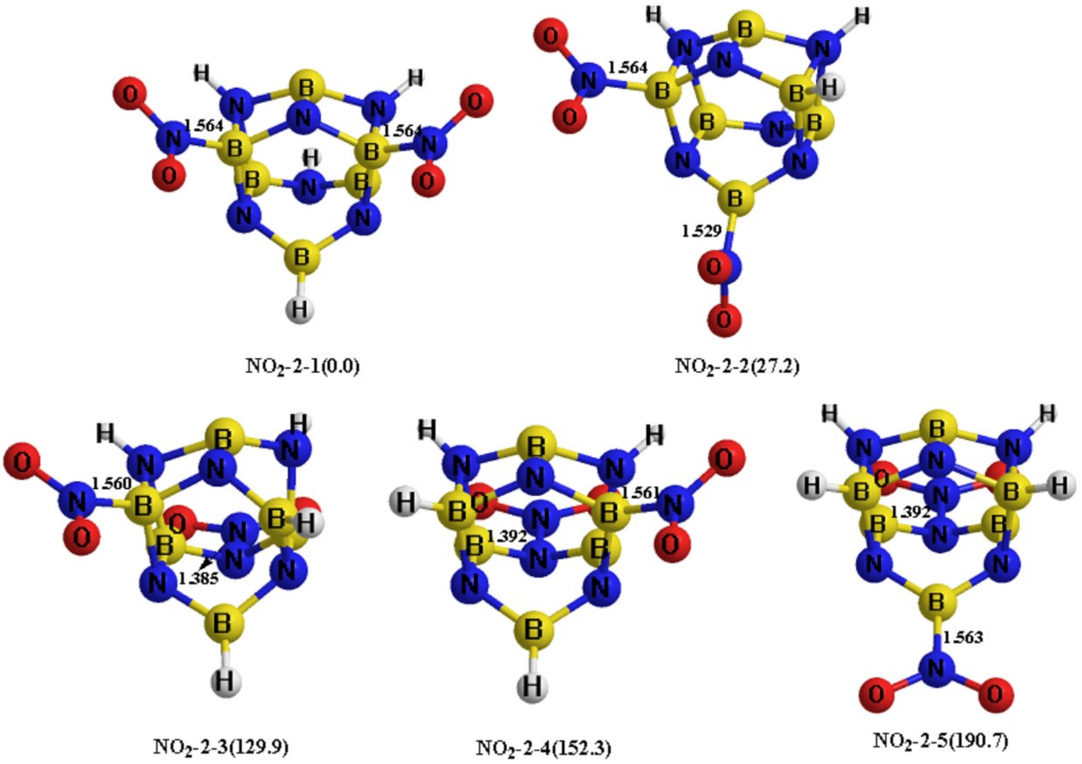

Figure S4 Optimized geometries (bond lengths in Å) at the M06-2X level for the dinitro-substituted BN-cage compoun6.5ds and their isomers. Relative energies (kJ mol^{-1}) at M06-2X level are indicated in parentheses

Table S3 Total energies (E in hartree), relative energies (ΔE in kJ/mol) and symmetries for the dinitro-substituted BN-cage compounds and their isomers at the M06-2X, ωB97XD and B3LYP level.

		2-1	2-2	2-3	2-4	2-5
M06-2X	E	-890.392507	-890.382149	-890.343073	-890.334532	-890.319916
	ΔE	0	27.2	129.9	152.3	190.7
	symmetry	Cs	C1	C1	C1	Cs
ω B97XD	E	-890.397972	-890.390197	-890.350567	-890.340891	-890.326487
	ΔE	0	20.4	124.5	149.9	187.8
	symmetry	Cs	C1	C1	C1	Cs
B3LYP	E	-890.389855	-890.382626	-890.341921	-890.331176	-890.318839
	ΔE	0	18.8	126.0	154.0	186.7
	symmetry	Cs	C1	C1	C1	Cs

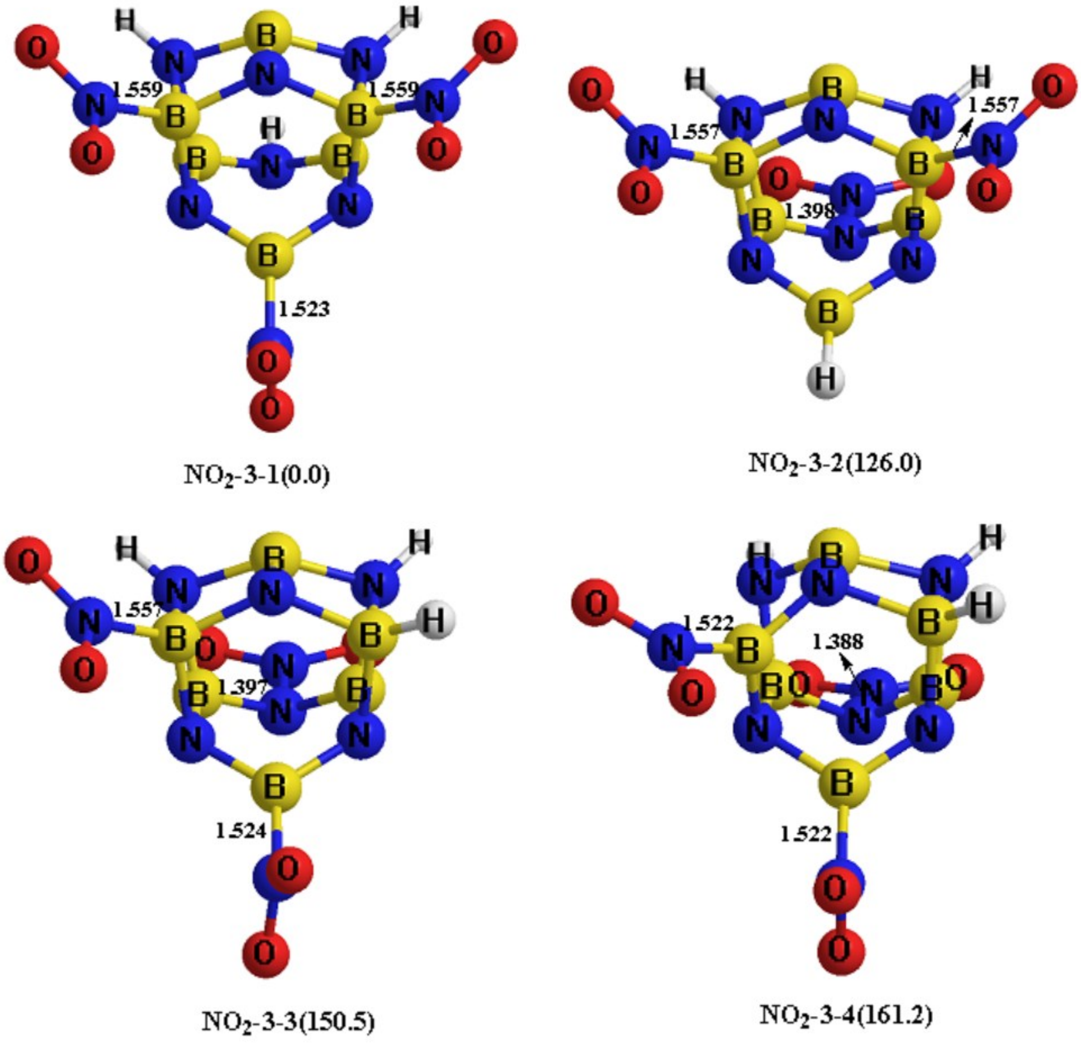

Figure S5 Optimized geometries (bond lengths in Å) at the M06-2X level for the trinitro-substituted BN-cage compounds and their isomers. Relative energies (kJ mol^{-1}) at M06-2X level are indicated in parentheses

Table S4 Total energies (E in hartree), relative energies (ΔE in kJ/mol) and symmetries for the trinitro-substituted BN-cage compounds and their isomers at the M06-2X, ωB97XD and B3LYP level.

		3-1	3-2	3-3	3-4
M06-2X	E	-1094.897765	-1094.849808	-1094.840454	-1094.836407
	ΔE	0	126.0	150.5	161.2
	symmetry	Cs	Cs	C1	C1
ω B97XD	E	-1094.913044	-1094.863675	-1094.856440	-1094.856044
	ΔE	0	129.7	148.7	149.7
	symmetry	Cs	Cs	C1	C1
B3LYP	E	-1094.898515	-1094.846713	-1094.840291	-1094.839262
	ΔE	0	136.0	152.8	155.7
	symmetry	Cs	Cs	C1	C1

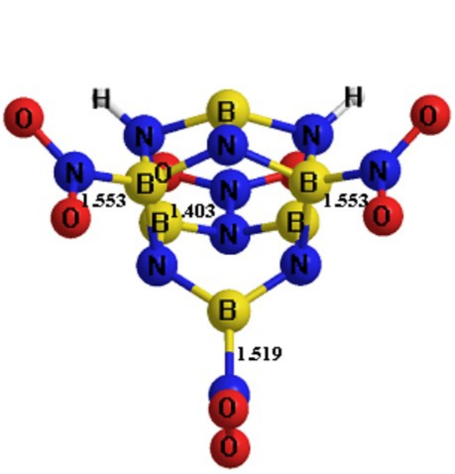

NO_2-4-1(0.0)

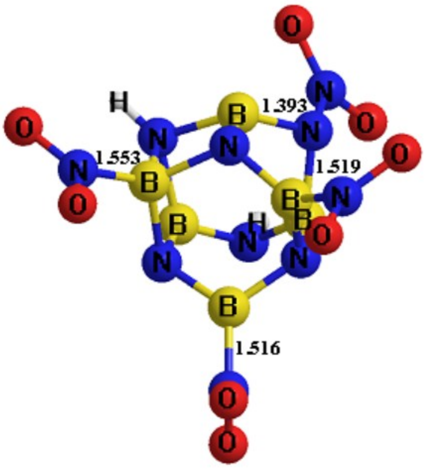

NO_2-4-2(8.8)

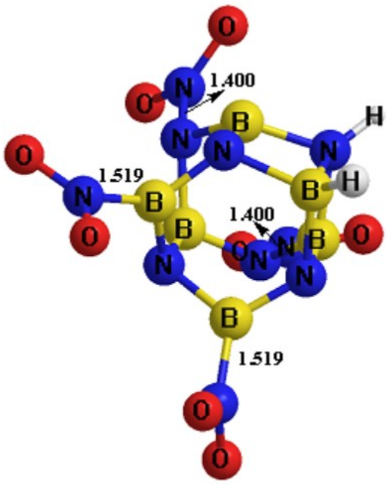

NO_2-4-3(188.6)

Figure S6 Optimized geometries (bond lengths in Å) at the M06-2X level for the tetranitro-substituted BN-cage compounds and their isomers. Relative energies (kJ mol^{-1}) at M06-2X level are indicated in parentheses

Table S5 Total energies (E in hartree), relative energies (ΔE in kJ/mol) and symmetries for the tetranitro-substituted BN-cage compounds and their isomers at the M06-2X, ωB97XD and B3LYP level.

		4-1	4-2	4-3
M06-2X	E	-1299.351602	-1299.348266	-1299.279785
	ΔE	0	8.8	188.6
	symmetry	Cs	C1	Cs
ωB97XD	E	-1299.385229	-1299.386115	-1299.308946
	ΔE	0	-2.3	200.4
	symmetry	Cs	C1	Cs
B3LYP	E	-1299.351958	-1299.351286	-1299.283357
	ΔE	0	1.7	180.0
	symmetry	Cs	C	Cs

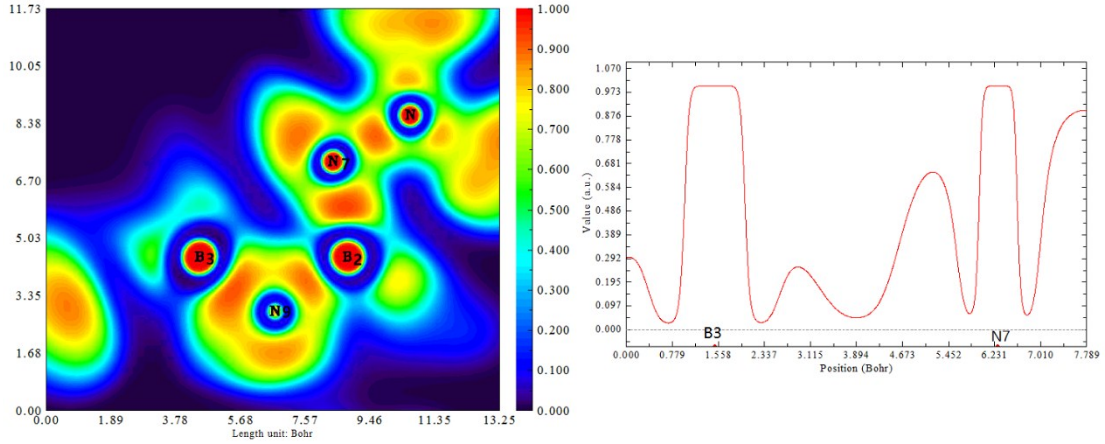

Figure S7 The color-filled map and curve map of ELF for B3-N7 bond path of NO_2-4-2 at the M06-2X levels of theory

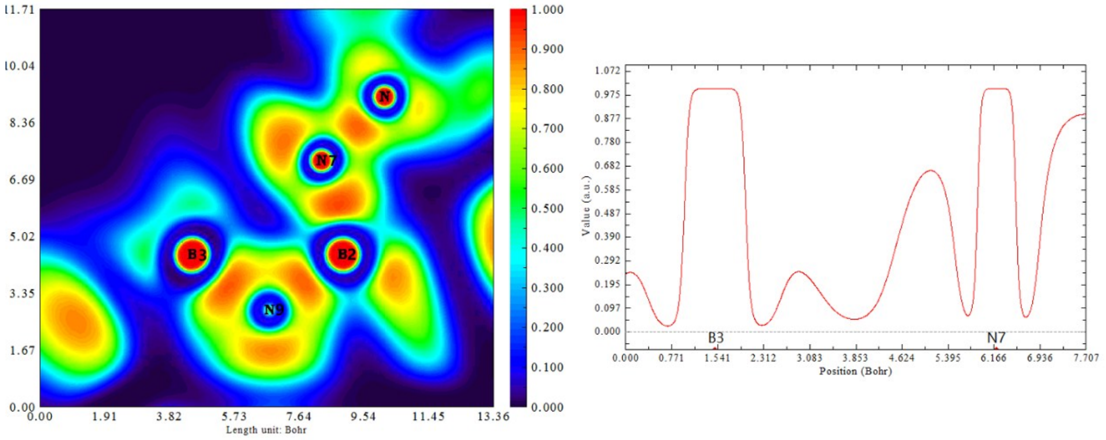

Figure S8 The color-filled map and curve map of ELF for B3-N7 bond path of NO_2-4-3 at the M06-2X levels of theory

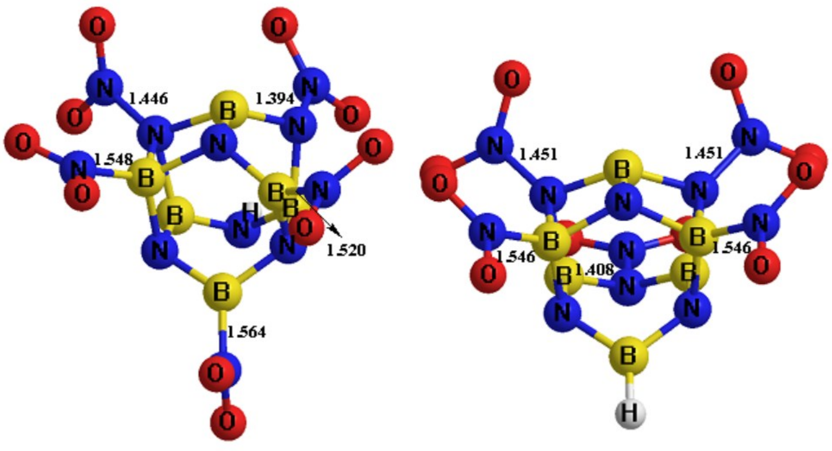

NO_2-5-1(0.0)　　　　NO_2-5-2(210.8)

Figure S9 Optimized geometries (bond lengths in Å) at the M06-2X level for the pentanitro-substituted BN-cage compounds and their isomers. Relative energies (kJ mol^{-1}) at M06-2X level are indicated in parentheses

Table S6 Total energies (E in hartree), relative energies (ΔE in kJ/mol) and symmetries for the pentanitro-substituted BN-cage compounds and their isomers at the M06-2X, ωB97XD and B3LYP level.

		5-1	5-2
M06-2X	E	-1503.799129	-1503.718869
	ΔE	0	210.8
	symmetry	C1	Cs
ω B97XD	E	-1503.829668	-1503.750364
	ΔE	0	208.3
	symmetry	C1	Cs
B3LYP	E	-1503.781134	-1503.709551
	ΔE	0	188.0
	symmetry	C1	Cs

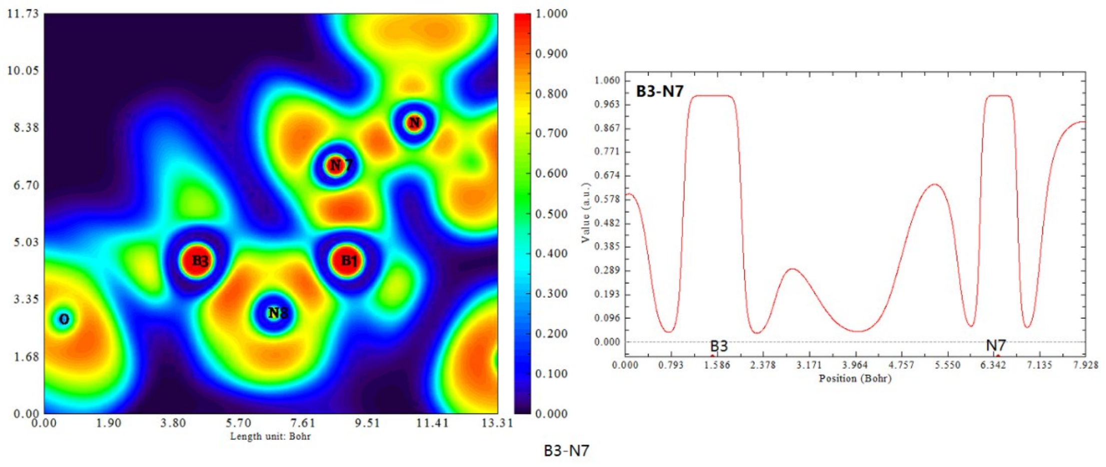

Figure S10 The color-filled map and curve map of ELF for B3-N7 bond path of NO_2-5-1 at the M06-2X levels of theory

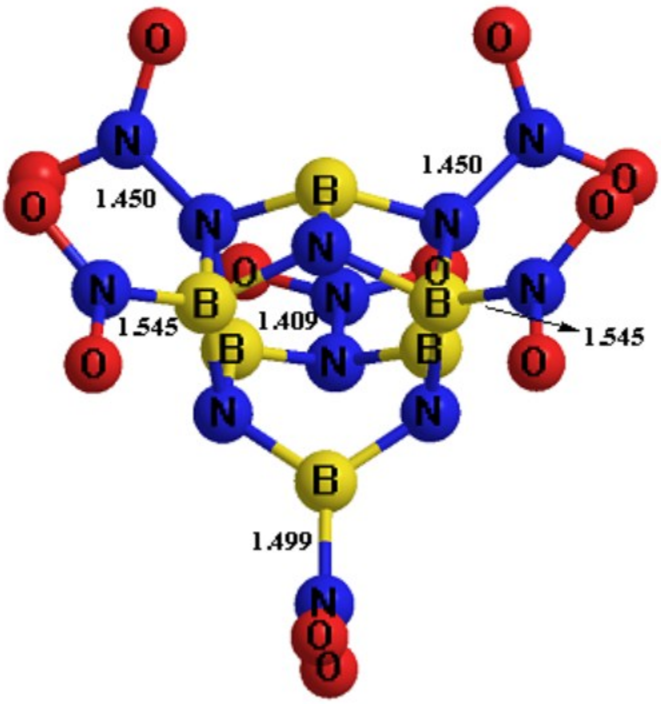

NO_2-6-1S(0.0)

Figure S11 Optimized geometries (bond lengths in Å) at the M06-2X level for the hexanitro-substituted BN-cage compounds and their isomers. Relative energies (kJ mol^{-1}) at M06-2X level are indicated in parentheses

Table S7 Total energies (E in hartree), relative energies (ΔE in kJ/mol) and symmetries for the hexanitro-substituted BN-cage compounds and their isomers at the M06-2X, ωB97XD and B3LYP level.

		6-1
M06-2X	E	-1708.236006
	ΔE	0
	symmetry	Cs
ω B97XD	E	-1708.272682
	ΔE	0
	symmetry	Cs
B3LYP	E	-1708.209609
	ΔE	0
	symmetry	Cs

Table S8 Total energies (E), zero point energies (ZPE), thermal correction values (H_T), heat of formation (kJ/mol), molecular surface properties(As, νσtot2), enthalpy of sublimation ($\Delta H°_{sub}$) and solid phase enthalpy of formation ($\Delta_f H°(s)$) of the NO_2-substituted BN-cage compounds at the M06-2X/6-311++G** level.

compounds	E (Hartree)	ZPE (kj/mol)	H_T (kj/mol)	As	νσtot2	$\Delta H°_{sub}$ (kj/mol)	$\Delta_f H°(s)$ (kj/mol)
NO2-1-1	-685.872	332.772	362.968	195.450	102.097	124.900	-289.545
NO2-2-1	-890.393	343.310	380.750	222.400	88.958	132.842	-449.511

Table S9 Total energies (E), zero point energies (ZPE), thermal correction values (H_T), heat of formation (kJ/mol), molecular surface properties(As, νσtot2), enthalpy of sublimation ($\Delta H°_{sub}$) and solid phase enthalpy of formation ($\Delta_f H°(s)$) of the NO_2-substituted BN-cage compounds at the ωB97XD/6-311++G** level.

compounds	E (Hartree)	ZPE (kj/mol)	H_T (kj/mol)	As	νσtot2	$\Delta H°_{sub}$ (kj/mol)	$\Delta_f H°(s)$ (kj/mol)
NO2-1-1	-685.871	331.089	361.455	195.563	99.072	123.909	-269.297
NO2-2-1	-890.398	342.447	379.931	222,788	92.823	134.435	-413.859

Table S10 Total energies (E), zero point energies (ZPE), thermal correction values (H_T), heat of formation (kJ/mol), molecular surface properties(As, νσtot2), enthalpy of sublimation ($\Delta H°_{sub}$) and solid phase enthalpy of formation ($\Delta_f H°(s)$) of the NO_2-substituted BN-cage compounds at the B3LYP/6-311++G** level.

compounds	E (Hartree)	ZPE (kj/mol)	H_T (kj/mol)	As	νσtot2	$\Delta H°_{sub}$ (kj/mol)	$\Delta_f H°(s)$ (kj/mol)
NO2-1-1	-685.869	325.331	355.989	198.391	101.246	125.908	-274.313
NO2-2-1	-890.390	333.32	371.382	227.201	95.520	137.619	-554.623
